# Psychiatric and psychosocial characteristics of suicide completers: A 13-year comprehensive evaluation of psychiatric case records and postmortem findings

**DOI:** 10.1192/j.eurpsy.2021.2264

**Published:** 2022-01-24

**Authors:** C. McMorrow, D. Nerney, N. Cullen, J. Kielty, A. VanLaar, M. Davoren, L. Conlon, C. Brodie, C. McDonald, B. Hallahan

**Affiliations:** 1 Centre for Neuroimaging & Cognitive Genomics (NICOG), Clinical Neuroimaging Laboratory, NCBES Galway Neuroscience Centre, College of Medicine Nursing and Health Sciences, National University of Ireland Galway, Galway, Ireland; 2 Department of Psychiatry, University Hospital Galway, Galway, Ireland; 3 Department of Pathology, University Hospital Galway, Galway, Ireland

**Keywords:** Drowning, hanging, schizophrenia, suicide, treatment nonconcordance

## Abstract

Currently, there are limited data comparing demographic and clinical characteristics of individuals who died by probable suicide and who did and did not previously attend mental health services (MHSs). This study compared demographic and clinical factors for both groups, in a Western region of Ireland over a 13-year period. Postmortem reports between January 1, 2006 and March 31, 2019 were reviewed for 400 individuals who died by probable suicide. Relevant sociodemographic and clinical data were extracted from individuals’ lifetime case notes. One hundred and fifty nine individuals (40%) had attended MHSs at some stage (“attendee”). Hanging was the most common method of suicide (61%), followed by drowning (18%) for both attendees and nonattendees of MHSs, with more violent methods utilized overall by nonattendees (*p* = 0.028). Sixty-eight percent of individuals who previously attempted hanging subsequently died utilizing this method. A higher proportion of attendees were female compared to nonattendees of MHSs (28.9 vs. 14.5%, *p* = 0.001). Recurrent depressive disorder (55%) was the most common diagnosed mental health disorder. For individuals with a diagnosis of schizophrenia, 39% had antipsychotic medications detectable in their toxicology reports. In conclusion, the majority of people who died by probable suicide had never had contact with MHSs, and nonattendees overall were more likely to utilize violent methods of suicide. Nonconcordance with psychotropic medications in psychotic patients and previous hanging attempt were highlighted as potential risk factors for death by probable suicide.

## Introduction

The World Health Organization estimates that 800,000 individuals die by suicide every year worldwide [1], with suicide the second highest cause of death among individuals aged 15–29 years globally. In Ireland, approximately 500 people (447–579) are reported to have died by suicide on an annual basis between 2000 and 2017, resulting in a suicide rate of 10.34–13.49 per 100,000 [2] with an approximate 4:1 ratio of male to female deaths noted (3.65–4.50).

Ascertaining putative causes for death by suicide has revealed a large array of potential clinical and psychosocial risk factors, which will be considered in this study. Psychological autopsy studies on completed suicides have reported that 60–90% of individuals experience a mental illness prior to their death by suicide, with mood disorders being most prevalent [3,4]. Alcohol and/or psychoactive substance misuse [3,5], a previous suicide attempt, and a history of deliberate self-harm (DSH) [6] are other well-documented significant risk factors associated with an increased risk of suicide.

Psychosocial factors implicated with increasing the risk of suicide include individuals experiencing childhood abuse [7,8], “single” marital status [9], and unemployment [10]. For individuals who die by suicide in Ireland, death by hanging has consistently been demonstrated as the most common method used [11,12]; however, methods of suicide utilized by individuals are related to multiple factors including gender, geographical location, cultural background, and method availability. More violent methods of suicide, including hanging and firearm use, have to date been more frequently utilized by males [13].

Although previous research has focused on demographic and clinical factors of suicide in attendees of mental health services (MHSs), there is limited data comparing these factors between attendees and nonattendees of MHSs, with the benefit of such research potentially highlighting psychosocial and clinical risk factors more specific for one cohort compared to the other. Therefore, the aim of this study was to comprehensively investigate the demographic and clinical characteristics of individuals who died by probable suicide (defined here as a death deemed to have resulted from intentional self-harm) in a Western region of Ireland over a 13-year period, and analyze group differences between individuals who have attended MHSs compared to those who have not. This study expands on previous research by Kielty et al. [12], which examined data on individuals who died by probable suicide and who attended MHSs from the same region over the initial 6 years of data collection. We sought to examine postmortem reports of all probable suicides (attendees and nonattendees) to determine the method of death and toxicology status at the time of death, and to cross-check these with psychiatric case records of attendees in order to examine and build upon current research of suicide statistics regarding both clinical and psychosocial risk factors for suicide.

## Materials and Methods

### Design and participants

All postmortem reports conducted at University College Hospital Galway (UCHG) between January 1, 2006 and March 12, 2019 were examined by the authors (C. McMorrow, C. McDonald, D.N., N.C., and J.K.) to identify individuals who died by probable suicide, defined here as a suicidal act with intent to die and evidence of self-inflicted injury where the outcome resulted in death [14]. Postmortem reports included individuals who died in a Western region of Ireland, including Galway City, and the surrounding more rural regions of Galway and Roscommon, which incorporated a population of 322,602 [15]. Postmortem examinations pertaining to all deaths in Galway City are conducted at UCHG, with approximately 75% of deaths from rural regions of Galway and Roscommon also conducted at UCHG and 25% undertaken at other locations. A senior psychiatrist (B.H./M.D.) and advanced nurse practitioner or psychologist (A.v.L., C. McMorrow, or D.N.) jointly determined based on postmortem records if individuals had died by “probable suicide.” In-depth details recorded on postmortem reports by a consultant pathologist were collected for the purposes of this study, including toxicology screenings (detailing alcohol levels, the presence of psychoactive substances, and medication levels), with police reports and witness statements also examined where available. Demographic data including age, gender, and marital status for individuals were collected. Only postmortems of individuals over the age of 18 were included in this study. Important considerations in determining if a death was due to probable suicide included the potential method associated with the death of the individual, toxicology screen (drug/alcohol levels in toxic range), and other factors detailed in police reports such as the presence of a suicide note or text. A conservative determination of whether suicide had occurred or not was adopted, with accidental death presumed where uncertainty remained in relation to the cause of death (e.g., a single-vehicle car crash resulting in death without positive toxicology or presence of a suicide note was not considered suicide).

Where it was determined that death was by probable suicide, computerized medical databases for the associated MHSs were examined to ascertain which individuals had previously attended these three MHSs. Attendance was considered any contact with a mental health clinician, and this includes those who may have had a single assessment as well as those who engaged with a care plan, thus throughout this paper we use the term “attendee” rather than service user to describe this cohort. The complete (paper) mental health clinical records were subsequently examined for individuals from Galway City and County, but were not available for individuals from Roscommon (*n* = 42). Data attained included additional demographic data such as employment or vocational status. Clinical data recorded included the total number of hospital admissions including their status (voluntary or involuntary) to MHSs, psychiatric diagnoses, the date (where applicable) of the individuals’ most recent discharge from hospital, and the last documented engagement with MHSs and treatments received. Psychiatric diagnoses according to treating clinician were based in accordance with the International Classification of Mental and Behavioral Disorders 10 (ICD-10) diagnostic criteria including diagnoses of alcohol and/or psychoactive substance abuse.

Ethical approval was attained prior to commencement of this study from the National University of Ireland Galway, School of Psychology, and Galway University Hospitals clinical research ethics committees.

### Statistical analysis

Statistical analysis was performed using the Statistical Package for Social Sciences (SPSS) 26.0 for Windows (SPSS Inc., IBM, New York). For clinical characteristics and toxicology data, as well as previous suicide attempts and DSH, we utilized simple ratios and percentage calculations. Sociodemographic variables were compared between those who did and did not attend MHSs utilizing the independent Student *t*-test for parametric data (clinical and sociodemographic data), and the *χ* ^2^ test or, where there was a small sample size, the Fishers’ exact test for nonparametric data.

## Results

### Sociodemographic and clinical data: entire cohort

Four hundred and thirteen individuals were determined to have died by probable suicide based on the above screening mechanisms; however, insufficient data were available on 13 individuals due to ongoing postmortem and pathological investigations, resulting in their exclusion. Of the remaining 400 individuals residing within the Health Service Executive (HSE) region of interest, 159 had previously attended the MHSs (40%).

Demographic data pertaining to all individuals and clinical data pertaining to attendees of MHSs who died by probable suicide are presented in Table 1. Overall, 79.8% of individuals were male, and the mean age of death was 42.1 (SD = 16.2) years. A higher proportion of females died by probable suicide in attendees compared to nonattendees of MHSs (28.9 vs. 14.5%, *χ* ^2^ = 12.314, *p* = 0.001). There was no statistical difference noted between attendees and nonattendees of MHSs pertaining to age of death (43.6 years [SD = 14.9] vs. 41.2 years [SD = 17.0], *t* = 1.44, *p* = 0.15); employment; or marital status, with single status (attendees = 59.3%, nonattendees = 56.2%, *χ* ^2^ = 6.954, *p* = 0.291) most common in both cohorts (Table 1).

**Table 1.** Demographic factors for attendees and nonattendees of mental health services (MHSs).

	Attendees of MHSs	Nonattendees of MHSs	Statistics
	*n* (%)	*n* (%)	*χ* ^2^, *df*, *p*
Gender			12.314, 1, 0.001
Male	113 (71.1)	206 (85.5)	
Female	46 (28.9)	35 (14.5)	
Marital status^a^			5.256, 3, 0.154
Single	86 (59.3)	80 (57.9)	
Married	29 (20.0)	38 (27.5)	
Divorced/separated	26 (17.9)	15 (10.2)	
Widowed	4 (2.8)	6 (4.4)	
Employment status^a^			3.538, 3, 0.316
Employed	63 (46.2)	39 (50.6)	
Unemployed	46 (33.8)	30 (39.0)	
Retired	17 (12.5)	6 (7.8)	
Third-level education	10 (7.4)	2 (2.6)	
Violence of method of death			4.845, 1, 0.028
Yes	132 (83.0)	218 (90.5)	
No	27 (17.0)	23 (9.5)	
Method of death			9.298, 4, 0.054
Hanging	91 (57.2)	154 (63.9)	
Drowning	30 (18.9)	40 (16.6)	
Overdose	26 (6.4)	19 (7.9)	
Gunshot	6 (3.8)	18 (7.5)	
Other	6 (3.8)	10 (4.1)	
Alcohol level (mmol/L)			4.413, 3, 0.220
0	106 (66.7)	144 (59.8)	
1–79	18 (11.3)	27 (11.2)	
80–199	25 (15.7)	40 (16.6)	
200+	10 (6.3)	30 (12.4)	
Positive toxicology for illicit substances			0.381, 1, 0.537
Yes	7 (4.4)	14 (5.8)	
No	152 (95.6)	227 (94.2)	

a Data are not available on all participants.

**Figure 1.**
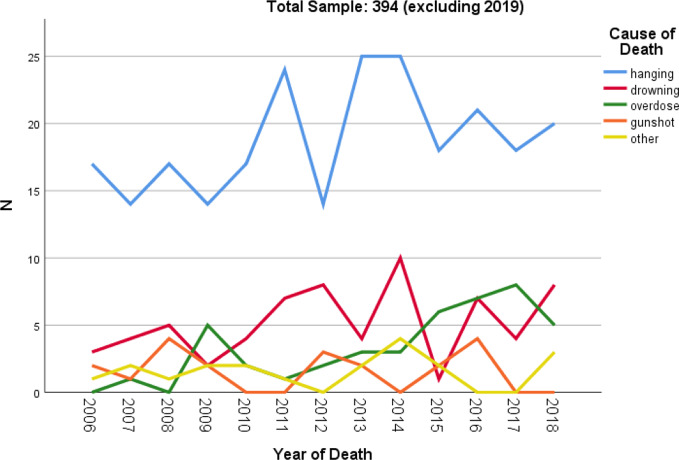
Method of death utilised for entire cohort of probable suicide for entire cohort.^a^ ^a^ 2019 was excluded from this section due to only 3 months of available suicide data.

The most common method of death was hanging (*n* = 245, 61.3%), with this method more commonly utilized by men compared to women (64.3 vs. 49.4%, *χ* ^2^ = 6.685, *p* = 0.01) with death by overdose more common in women compared to men (19.8 vs. 9.1%, *χ* ^2^ = 7.756, *p* = 0.005) (Table 2). Hanging was consistently the most common method utilized for suicide between 2006 and 2018 among both nonattendees (63.9%) and attendees (57.2%) of the MHSs (Figure 1), with both groups predominantly utilizing violent methods of death, that is, hanging (61%), drowning (18%), and gunshot (6%), with this statistically more likely in nonattendees of the MHSs (90.5 vs. 83.0%, *χ* ^2^ = 4.845, *p* = 0.028).

**Table 2.** Gender and demographic data.

	Male		Female		Statistics
	*n*	(%)	*n*	(%)	*χ* ^2^, *df*, *p*
Age^a^					1.368, 2, 0.505
18–34	111	(34.6)	27	(32.9)	
35–54	144	(45.4)	33	(40.2)	
55+	64	(19.8)	21	(25.6)	
Violent method					8.777, 1, 0.003
Yes	287	(90.0)	63	(77.8)	
No	32	(10.0)	18	(22.2)	
Method					15.767, 4, 0.003
Hanging	205	(64.3)	40	(49.4)	
Drowning	50	(15.7)	20	(24.7)	
Overdose	29	(9.1)	16	(19.8)	
Gunshot	23	(7.2)	1	(1.2)	
Other^b^	12	(3.8)	4	(4.9)	
Alcohol level (mmol/L)					3.958, 3, 0.267
0	192	(60.2)	58	(71.6)	
1–79	37	(11.6)	8	(9.9)	
80–199	55	(17.2)	10	(12.3)	
200+	35	(11.0)	5	(6.2)	
Positive toxicology for illicit substances					0.174, 1, 0.677
Yes	16	(5.0)	5	(6.2)	
No	303	(95.0)	76	(93.8)	

a Age is unavailable for one participant.

b Other methods include poisoning, RTA, self-mutilation, and self-asphyxiation.

No ethanol was detected in 250 (62.5%) individuals. High levels of ethanol (>200 mmol/L) were found in 40 (10.0%) individuals, with no statistical differences in ethanol levels between attendees and nonattendees of MHSs (*χ* ^2^ = 4.413, *p* = 0.22; Table 1).

### Clinical data for mental health service attendees

The clinical characteristics of the 159 individuals who died by probable suicide and who previously attended MHSs are detailed in Table 3. The most common psychiatric diagnosis noted in the clinical records was recurrent depressive or major depressive disorder (*n* = 87, 54.7%), followed by alcohol dependence syndrome or harmful use of alcohol (*n* = 32, 20.1%), with 16 individuals (10.1%) having both diagnoses.

**Table 3.** Clinical characteristics and toxicology data of attendees of mental health services.

Variable	*n*	%
Psychiatric diagnosis^a^		
Major or recurrent depressive disorder	87	54.7
Alcohol use disorder	32	20.1
Personality disorder	8	5.0
Schizophrenia	13	8.2
Bipolar disorder	14	8.9
An anxiety disorder	22	13.9
Eating disorder	3	1.9
Positive toxicology^b^	88	55.3
Antidepressants	55	62.5
Antipsychotics	30	34.0
Benzodiazepines	36	40.9
Opiates	9	10.2
Hypnotics	27	30.7
Pain killer	18	20.5
Engagement with MHS		
Low (<5 contacts)	81	51.0
Moderate (15–19 contacts)	17	10.7
High (20–29 contacts)	4	2.5
Very high (30+ contacts)	13	8.2
No episodes on file	44	27.7
Duration before death since last psychiatric contact		
Inpatient	3	2.6
<2 weeks	15	13.2
2–4 weeks	5	4.4
4 weeks–3 months	25	21.9
3–6 months	14	12.3
6–12 months	14	12.3
>12 months	23	20.2
>5 years	15	13.2
Treatment adherence	10	16.7
Previous suicidality		
History of suicide talk	75	47.2
History of deliberate self-harm	82	51.6
History of a suicide attempt^a^		
0	70	44.0
1	41	25.8
2	31	19.5
3+	17	10.7

a Not every attendee had a documented diagnosis or a diagnosis that could be accurately provided retrospectively. Dual diagnoses included.

b Where more than one type of drug from the same class (i.e., selective serotonin reuptake inhibitor and tricyclic antidepressants) appeared in toxicology screens, it was recorded as 1 class per individual.

Limited previous engagement (<5 interactions) with the MHSs was noted in 81 individuals (70.4%) for whom data were available. Previous rates of engagement in the MHSs (≤5 vs. >5 interactions) were not associated with gender (*χ* ^2^ = 0.592, *df* = 1, *p* = 0.44) or age (*t* = 0.070, *p* = 0.94).

Full clinical records providing detailed information pertaining to clinical interactions and recent contact with MHSs were available for 114 (71.7%) individuals. Seventy-six individuals (66.7%) had contact with the MHSs within a year of their death, with 20 individuals (17.5%) having contact in the 4-week period prior to their death and a further 3 individuals (2.6%) dying in in-patient care.

### Previous suicide attempts for mental health service attendees

At least one previous suicide attempt had been undertaken by 87 individuals (54.7%), with 17 individuals (10.7%) having three or more previous suicide attempts. The most common previous suicide attempt was attempted overdose (*n* = 87, 54.7%) followed by attempted hanging (*n* = 29, 17.1%). Nineteen individuals (67.9%) subsequently died by hanging who had a previous documented attempt utilizing this method (Table 4). A history of DSH (without intent to end life) was present in 82 individuals (51.6%), with 73 individuals (45.9%) having a history of both a suicide attempt and a history of DSH.

**Table 4.** Previous suicide attempts and deliberate self-harm in patients who died by probable suicide.

Method of death	History of previous suicide attempt(s)		Method of death same as a previous method employed in a suicide attempt		Attempted hanging	Attempted drowning	Attempted overdose	Attempted cutting	Attempted poisoning	Attempted other	No attempt
	*n*	(%)	*n*	(%)	*n*	*n*	*n*	*n*	*n*	*n*	*n*
Hanging (*n* = 91)	29	17.1	17	41.5	**19**	4	42	10	5	7	40
Drowning (*n* = 30)	18	10.6	5	12.1	2	**4**	17	5	0	0	17
Overdose (*n* = 26)	87	51.2	17	41.5	6	1	**24**	4	1	1	6
Other (*n* = 12)	36	21.1	2	4.9	2	9	4	3	0	0	7
Total (*n* = 159)	170		41		29	18	87	22	6	8	70

Individuals who made three or more suicide attempts were more likely to have a previous forensic history, a history of alcohol or psychoactive substance use compared to individuals with two or less previous suicide attempts.

### Toxicology reports

Antidepressant medications were detected on toxicology testing in 103 individuals (25.8%), followed by benzodiazepines (*n* = 54, 13.5%) and antipsychotic medications (*n* = 45, 11.3%). For individuals who had attended MHSs with a documented history of a major depressive or recurrent depressive disorder, 34 individuals (51.5%) had antidepressants detectable on toxicology screening. Of the 13 individuals with a diagnosis of schizophrenia, 5 (38.5%) had antipsychotic medications detectable on toxicology screening.

## Discussion

In this study, we evaluated demographic data and postmortem reports of 400 individuals who died by probable suicide over a 13-year period in a region in the West of Ireland and included both attendees and nonattendees of MHSs, with comparative analysis on a range of sociodemographic and clinical factors undertaken between these two groups.

Forty percent of individuals had some previous contact with MHSs in their lifetime, which is consistent with some previous findings [16,17]. The lack of contact with MHSs for 60% of individuals does not indicate no contact with any service for mental health difficulties for all these individuals, as primary care, addiction, or other counseling services, may have been assessed. Indeed, 19% of nonattendees had antidepressants present in their toxicology screen, suggesting that at least a minority of patients were treated with pharmacological agents in primary care or private practice (private psychologist/psychiatrist) for mental health-related disorders.

### Diagnoses

A previous diagnosis of a major depressive disorder was present in over half of individuals that died by probable suicide according to clinical records, a finding consistent with several previous European studies [18], but is in contrast to some previous findings in studies conducted in Asia and Australia [11,19], that found psychotic disorders, such as schizophrenia, the most prevalent diagnosis in individuals who died by suicide. Only 9% of this cohort had a diagnosis of a schizophrenia spectrum disorder, and of these individuals, just one-third were taking prescribed medications. This suggests a high nonconcordance rate with medications prior to their death. Alcohol or substance use disorders were the second most frequent diagnosis in this study, a finding which is also consistent with a number of previous findings [20], with 10% of individuals having both alcohol use and major depressive disorders, a finding associated with an increased risk of suicide [21]. It is possible in some instances that alcohol consumption was linked to the social theory of individual’s attempting to self-medicate to better cope with unpleasant emotions (including significant depressive symptoms) or difficult psychosocial situations [22]. However, toxicology reports noted that only 10% of individuals had high levels of alcohol detectable. Our figures of alcohol use prior to an individuals’ death by probable suicide are similar to some previous studies [23]. Thus, although alcohol can have a disinhibiting effect and increase the risk of someone attempting suicide or engaging in self-harm [24], no firm association between probable suicide and alcohol intoxication was demonstrated in this study.

### Sociodemographic data

Clinical and demographic features were similar between attendees and nonattendees of MHSs, with the exception of gender. Our finding of a 4:1 male to female death by probable suicide for the entire cohort is consistent with previous research findings [12,21]. However, attendees of MHSs had a lower male to female ratio of 2.5:1 for suicides, potentially demonstrating that males attend or seek input from MHSs less frequently, which may be due to perceptions of societal expectations and traditional gender roles, wherein males are less likely to discuss or seek help for their mental health problems [25,26]. Other sociodemographic features that were overrepresented in individuals who died by probable suicide in this study compared to the general population, but did not differ between attendees and nonattendees of MHSs, included being single, unemployed, and residing alone, and are consistent with previous research findings [17].

### Method of suicide

Death by hanging was consistently the most prevalent method of suicide followed by either death by drowning or overdoses. Death by hanging has consistently been demonstrated to be the most common method of death by suicide in Ireland and in many other jurisdictions [4,11,12]. Consistent with existing literature, males were significantly more likely to use violent methods such as hanging, whereas females were more likely to utilize less violent methods such as overdose of medications [13]. Nonattendees were significantly more likely to die by more violent methods of suicide compared to attendees of MHSs. Relatively high rates of suicide by drowning (17.5%) were noted in this study compared to some previous findings, particularly in females (25%). Studies in Europe and Australia, for example, have noted rates of suicide secondary to drowning accounting for <7% deaths [27]. Methods of suicide can be related to multiple factors including geographical location and one probable reason for this relatively high rate of probable suicide by drowning pertains to the location of Galway, which is adjacent to the Atlantic Ocean, with a fast-flowing river running through the city itself, with several lakes additionally located in the region. A recent program has commenced to patrol bridges in the city, in an effort to reduce the suicide rate secondary to drowning, and the impact of this initiative will be evaluated in future research studies. Probable suicide secondary to fatal gunshot wounds accounted for 6% of deaths, a figure lower than in some jurisdictions where firearms are more easily accessible [28]. Despite this relatively low rate, stricter regulations pertaining to gun ownership including extensive psychological evaluations of individuals applying for gun licenses may be optimal, as gun licenses are still attainable in Ireland without psychological or psychiatric review. This may be particularly pertinent as more individuals without a previous history of engagement with the MHSs (albeit not a statistically significant finding) utilized this method (7.5 vs. 3.8%).

### Previous suicide attempts and recent engagement with mental health services

A history of a past suicide attempt(s) is a well-established risk factor for eventual death by suicide with 56% of our cohort having a history of suicide attempts (1 or more), with overdose accounting for the highest number of previous attempts. Over two-thirds of individuals who previously attempted hanging subsequently died utilizing this method, whereas individuals who subsequently died by other methods rarely had a previous attempt at hanging. This suggests that a previous attempt of hanging may be a risk factor for hanging in the future, which is in contrast to suicide attempts by other methods such as by overdose. This finding is consistent with our previous study [12], and a long follow-up study of a Swedish cohort [29], where 90% of individuals who utilized hanging in an “index attempt,” subsequently died by the same method. A history of DSH including nonfatal acts of DSH is another well-recognized risk factor for future death by suicide [6] and was also found in this study, with half of the participants (for whom data were available) having a documented history of DSH. Twenty percent of individuals had contact with MHSs (inpatient and outpatient) within 4 weeks of their death by probable suicide. This period of increased risk of suicide has previously been highlighted [30]. Our study demonstrated lower rates of suicide deaths occurring within 1 week of discharge compared to some previous studies [16], and potentially relates to patients being routinely contacted by MHSs in this region within 1–2 weeks after discharge from hospital.

## Limitations

There are a number of limitations with this study. First, complete psychiatry clinical records were unavailable for individuals who attended one of the MHSs resulting in a lack of availability of some data including previous suicide attempts. Second, as only clinical data of individuals known to MHSs were included, it is possible that those listed as nonattendees had a previous suicide attempt or possessed mental health difficulties which were only addressed by their general practitioner. It is also possible that while our clinical notes for attendees may have implied nonadherence to treatment medications, other healthcare providers outside of MHSs could have discontinued specific psychotropic medications. This is, however, unlikely for mental disorders, such as schizophrenia, where management is almost exclusively provided by MHSs. Thus, nonconcordance rates with pharmacological treatments stated in this study may not have been as high as stated. Third, no formal psychometric instruments were administered routinely to the individuals in this study; however, all diagnoses were based on ICD-10 diagnostic criteria as documented in the clinical notes, and were corroborated by the presence of documented symptoms of that diagnosis. All psychiatry clinical notes were comprehensively evaluated by three individuals including one experienced clinician (B.H.). Finally, although we distinguish between suicide attempts and DSH without an attempt to end one’s life, caution is required with such a distinction on the basis of psychiatric clinical notes alone.

## Conclusions

In this comprehensive study of all deaths by probable suicide in a defined region in the West of Ireland, the majority has never had contact with MHSs. Individuals most commonly died by hanging and drowning and nonattendees overall were more likely to utilize more violet methods of suicide. Although more males across both attendees and nonattendees of MHSs died by probable suicide; there was a higher proportion of females among attendees of MHSs. Previous attempts at hanging were particularly prevalent in individuals who subsequently died by this method. At the time of death, approximately one-half of individuals with a diagnosis of a major depressive disorder and two-thirds with a diagnosis of schizophrenia according to toxicology reports were not taking psychotropic medications prescribed for these disorders, suggesting a high rate of treatment nonconcordance in individuals who died by probable suicide.

## Data Availability

Data resources have not been made publicly available.
